# A large-scale chromosomal inversion is not associated with life history development in rainbow trout from Southeast Alaska

**DOI:** 10.1371/journal.pone.0223018

**Published:** 2019-09-20

**Authors:** Spencer Y. Weinstein, Frank P. Thrower, Krista M. Nichols, Matthew C. Hale

**Affiliations:** 1 Department of Biology, Texas Christian University, Fort Worth, United States of America; 2 Ted Stevens Marine Research Institute, Alaska Fisheries Center, NOAA, Juneau, AK, United States of America; 3 Conservation Biology Division, Northwest Fisheries Science Center, Seattle, WA, United States of America; National Cheng Kung University, TAIWAN

## Abstract

In studying the causative mechanisms behind migration and life history, the salmonids–salmon, trout, and charr–are an exemplary taxonomic group, as life history development is known to have a strong genetic component. A double inversion located on chromosome 5 in rainbow trout (*Oncorhynchus mykiss*) is associated with life history development in multiple populations, but the importance of this inversion has not been thoroughly tested in conjunction with other polymorphisms in the genome. To that end, we used a high-density SNP chip to genotype 192 F_1_ migratory and resident rainbow trout and focused our analyses to determine whether this inversion is important in life history development in a well-studied population of rainbow trout from Southeast Alaska. We identified 4,994 and 436 SNPs–predominantly outside of the inversion region–associated with life history development in the migrant and resident familial lines, respectively. Although F_1_ samples showed genomic patterns consistent with the double inversion on chromosome 5 (reduced observed and expected heterozygosity and an increase in linkage disequilibrium), we found no statistical association between the inversion and life history development. Progeny produced by crossing resident trout and progeny produced by crossing migrant trout both consisted of a mix of migrant and resident individuals, irrespective of the individuals’ inversion haplotype on chromosome 5. This suggests that although the inversion is present at a low frequency, it is not strongly associated with migration as it is in populations of *Oncorhynchus mykiss* from lower latitudes.

## Introduction

Understanding the genetic basis of adaptation is a central aim of both evolutionary biology and population genetics. Although populations are adapted to their environments, individuals from different populations with different genetic backgrounds can develop the same phenotype in response to similar environmental cues [1, 2]. The underlying genetic mechanisms leading to parallel adaptation varies between populations and includes selection operating on the same allele in the same locus, different alleles in the same locus, and different alleles in different loci [3–5]. Previous studies on the genetic basis of adaptive traits have mostly focused on phenotypes with simple patterns of inheritance–i.e., few alleles of large effect [3, 6, 7]. Although informative, such patterns are not consistent with respect to traits determined via complex patterns of inheritance–i.e., many alleles of small effect. Such traits may also show evidence of parallel adaptation (reviewed in [8]) and therefore, more studies investigating parallel adaptation in complex polygenic traits are required.

Chromosomal rearrangements (including inversions, deletions, translocations, fusions, and fissions) have been associated with the development of multiple adaptive phenotypes, including salinity tolerance and migratory movements in Atlantic cod (*Gadus morhua*; [9, 10]), crypsis in stick insects (*Timema cristinae*; [11]), and migratory behavior in rainbow trout (*Oncorhynchus mykiss*; [12]). Rearrangements have an effect on recombination in the affected region as homologous chromosomes in heterozygous individuals cannot recombine during meiosis. Thus, genes within chromosomal rearrangements regularly show high levels of linkage disequilibrium, as haplotypes within the rearrangement are inherited together. This facilitates the development of adaptive phenotypes, as co-adapted alleles can be shielded from the effects of recombination [13–15]. However, within a species, the frequency of inversions can vary between different populations, suggesting population-specific genetic effects [16].

Two large, adjacent inversion polymorphisms (spanning ~55MB and containing more than 1,000 protein-coding genes; [12]) associated with multiple adaptive traits–including development rate, sexual maturation and spawning date, and life history development [12, 17–23]–can be found on chromosome 5 (Omy05) of the rainbow trout genome. The link between the inversion polymorphisms and life history variation was originally proposed due to the high frequency of the inverted (or derived) form of the inversion in above barrier populations of resident rainbow trout from central California. Below barrier anadromous populations in the same region mostly lack the inversion (displaying the ancestral form; [12, 23, 24]. Subsequent investigations have found a strong latitudinal component to the inversion with an increased frequency of the derived chromosome arrangement with increased latitude [23, 25]. Across the rainbow trout range, however, both QTL and GWAS studies suggest that the genetic basis of life history development in rainbow trout has a strong population-specific component with different regions of the genome showing associations with life history (e.g., [26–29]). Therefore, it is important to not only investigate the association between the inversion on Omy05 and anadromy, but also to screen other regions of the genome to evaluate the relative importance of the inversion to loci throughout the genome that may influence life history development. Doing so will also allow an understanding of the importance of parallel versus population specific genetic effects in the development of migratory life history variation, allowing comparisons to be made to other species where the genetic bases for parallel adaption are well understood.

The Sashin Creek system on Baranof Island in southeastern Alaska has become a model population for studying the genetic basis of migration in rainbow trout, as previous quantitative genetic, QTL, and GWAS analyses all confirm a strong genetic basis to life history development (e.g., [27, 29, 30]). The Sashin Creek drainage is composed of a native, largely anadromous population in Sashin Creek, and a resident population in Sashin Lake that was founded by transplants from Sashin Creek (individuals originating below the two large barrier waterfalls that separate the creek and the lake; [31, 32]). Since founding, strong selective pressures against the production of migrants in the lake have resulted in the development of a largely resident lake population [29]. Herein, we present data generated from both resident and migrant rainbow trout produced from two experimental crosses that were analyzed with respect to two main aims: first, to investigate the presence and abundance of both forms of the chromosome 5 inversion and to test if the inversion is associated with life history adaptation, and second, to utilize large-scale genotyping to locate polymorphisms associated with the development of different life histories in two populations of rainbow trout subjected to different selective regimes.

## Materials and methods

### Sampling and SNP genotyping

Samples for this study came from two experimental crosses initiated in May 2010 in the Sashin Creek watershed in southeastern Alaska (see [33] for details). All experimental crosses were conducted with permission from Alaska’s Department of Fish and Game (permit number SF2010-221). All methods involving live rainbow trout (detailed below) were approved by Purdue University’s IACUC (protocol number 06–033). MS-222 (50 mg/L; Argent Chemicals, Redmond, WA) was used to non-lethally anesthetize samples before measuring and fin clipping for DNA analysis (details below). Twelve mature returning migrant trout were sampled at Sashin Weir (56.379: -134.653 (six males and six females)) and twelve mature resident trout from Sashin Lake (56.365: -134.679 (six males and six females)) were used to create six migrant-by-migrant (A x A) and six resident-by-resident (R x R) families, respectively. Migrants were caught in a weir trap at the outlet of Sashin Creek and residents were caught using baited hoop traps in Sashin Lake. Gametes were collected by applying light pressure on the abdomen, placed in zip lock bags, and kept at 4°C until fertilization on the same day at the Little Port Walter Marine Station. Post-fertilization, embryos were housed at the Little Port Walter Marine Station in the dark in circulating stack chambers supplied with ambient water from Sashin Creek. Upon reaching the swim-up stage (about 55 days post-fertilization), fish were housed in 170 L outdoor micro raceways split by cross-type (all A x A samples were housed in one raceway and all R x R samples were housed in one raceway). Again, water came directly from Sashin Creek. Fish were fed until satiation with a commercial trout fish food and were maintained under natural photoperiod, water temperature, and oxygen concentration. In June 2012, samples were non-lethally anesthetized and fork length, weight, and life history characterization were determined. Individual fish were categorized into four groups based on a combination of morphological features and the expression of gametes:

1. Migratory smolts were identified based on a lack of gamete expression, a condition factor (weight/length^3^ x 100,000) < 1.0, and the appearance of skin silvering (a morphological trait associated with migration);
2. Precocious mature resident trout were identified based on the expression of gametes and a lack of phenotypic signs associated with migration;
3. Immature resident rainbow trout were identified based on parr marks along the dorsal side of the individual and a lack of phenotypic signs associated with migration, failure to express gametes, and a condition factor > 1.0;
4. Indeterminate individuals were identified by a combination of features associated with anadromy and residency. For example, indeterminates had some signs of skin silvering and did not express gametes, but still retained parr marks and/or a pink stripe along the lateral line. Due to the inability to discern life history, indeterminates were left out of further analyses.

Fin clips were also taken from these samples for genetic analysis. DNA was extracted from fin clips using the Qiagen DNeasy Blood and Tissue DNA extraction kit (following manufacturer’s protocol; Qiagen, Hilden, Germany) and diluted to a standard concentration of 50 ng/μL. Given the known sex bias in life history, fish that had not reached sexual maturity were sexed with *OmyY1*, a marker on the male-specific portion of the Y-chromosome [34]. The sexing PCR protocol followed Brunelli et al. 2008 [34], except annealing conditions were 60°C for 50 seconds. Sex was determined by running 4 μL of PCR product on a 1.5% agarose gel stained with Gel Red and viewed under UV light.

A total of 192 individuals were genotyped on an Affymetrix 57K SNP chip ([35]; Neogen GeneSeek Operations, Lincoln, Nebraska). These individuals were selected based on life history, condition factor, sex, and familial origin. A total of 124 samples (38 male migrants, 37 female migrants, 38 mature males, and 11 female residents) were genotyped from the A x A line and 68 samples (16 male migrants, 18 female migrants, 24 mature males, and 10 female residents) were genotyped from the R x R line.

### Population genomic analyses

Although the positions of the markers on the Affymetrix chip were known, we re-aligned the loci against the newest version of the rainbow trout genome to increase the number of mapped SNPs (fewer than 2,000 SNPs could not be mapped compared to over 25,000 SNPs unable to be mapped when mapping against the old version of the rainbow trout genome [12], Genbank accession number GCA_002163495.1). Alignments were performed using Bowtie 2 with default parameters [36]. SNPs that could not be mapped and those that mapped to multiple positions were discarded. SNPs that were fixed within a cross were removed from analysis in that cross type.

Initially, a Principle Component Analysis was carried out in PLINK version 1.9 [37] to determine kinship for running a GLM between genotype and migratory tactic. All SNPs with a minor allele frequency less than 0.05 within a cross and SNPs with significant linkage disequilibrium (r^2^ > 0.1) were removed. Clusters were generated using the–*pca var-wts* command in PLINK 1.9 which produces eigenvalues for the first 20 principle components and loading scores for each marker. Loading scores suggested that a random distribution of alleles on the rainbow trout genome contributed to eigenvalues on principle components 1 and 2. Principle component plots were generated using ggplot2 in R. Identity-by-descent coefficients were calculated in PLINK using PCA coordinates with default parameters.

To identify markers associated with life history development we employed a GLM approach, using TASSEL, analyzing each cross separately [38] with kinship and sex as co-factors in the model. To identify markers significantly associated with life history, a Benjamini-Hochberg FDR correction was applied to the raw p-values to account for Type I error, and a corrected p-value threshold of 0.01 was used to infer significance. Additional population genetic statistics were collected per A x A and R x R cross types to investigate patterns of selection within the A x A and R x R groups. Tajima’s D (to compare allelic frequency patterns), observed heterozygosity, and expected heterozygosity were calculated within each group using TASSEL [38]. Nei’s pairwise *F*_*ST*_ was calculated between the two crosses using the R package ‘hierfstat’.

### Sliding window and outlier analyses

After identifying individual markers significantly associated with life history, we performed sliding window analyses to identify regions of the genome potentially under selection. The sliding window analyses were carried out within the R package ‘WindowScanR’; we performed these analyses using raw p-values, observed (*H*_*O*_) and expected (*H*_*S*_) heterozygosity, *F*_*ST*_, and Tajima’s D. For all statistics excluding Tajima’s D, we used a window size of 1 MB and a step size of 0.5 MB. For Tajima’s D, each window contained 50 markers with a step size of 25 markers. To identify outliers, samples from both crosses were combined and compared to find alleles that might be targets of selection between resident and migratory populations. A test for loci displaying signals of divergent selection (outlier loci) was conducted with BAYESCAN 2.1 [39] using all non-monomorphic loci and default parameters (prior odds: 10), and a false discovery rate of 0.05.

### Genome alignment and marker annotation

To determine whether markers associated with life history development were located within genes of known function, we performed a BLAT analysis (maximum e-value of 1e-10) to map an annotated rainbow trout transcriptome [40] to the new version of the rainbow trout genome. Positional information was inferred if an annotated transcript matched a unique genomic position. Genes encompassing SNPs associated with life history development were grouped based on their functionality as inferred by gene ontology terms, from the Gene Ontology database, associated with the protein-coding gene. For this analysis, we analyzed the crosses both separately and together to see if any genes or regions of the genome appeared to be associated with life history regardless of cross. A limitation of this analysis was the inability to map every transcriptome contig back to the genome, due to a combination of a relatively recent salmonid whole genome duplication (causing tetraploid regions of the *O*. *mykiss* genome; [41]) and possible incomplete gene annotations.

### Linkage disequilibrium analysis

PLINK v1.9 was used to determine patterns of linkage disequilibrium (LD) for each chromosome. Samples were split by cross type and analyzed separately. Patterns of LD were determined for each chromosome within each cross type separately using the following command–*chr-set 29 no-xy no-mt–r2 inter-chr–chr* where r^2^ is the measurement of LD, inter-chr allows for measurements of LD to be made between distant markers, and–chr allows for the selection of a specific chromosome. Resulting values of r^2^ were analyzed in ggplot2 in R to investigate patterns of recombination.

## Results

### Markers associated with anadromy

**Fig 1:**
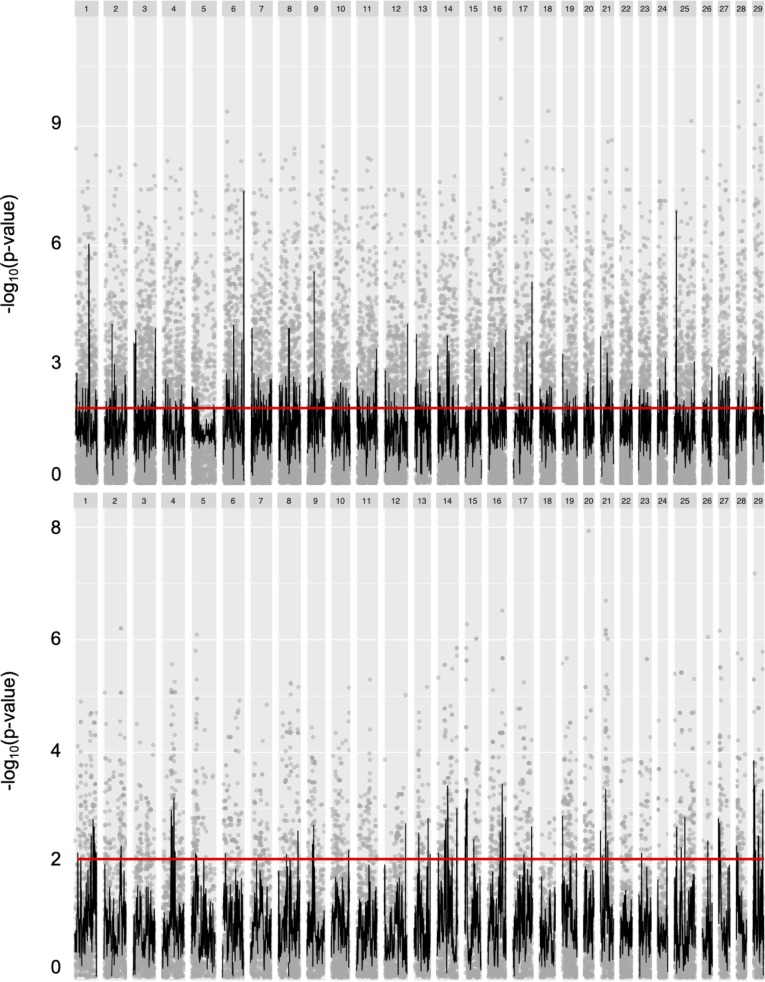
Sliding window analysis of raw p-values (-log_10_ transformed) from association tests between migrants and residents within the A x A cross (top) and the R x R cross (bottom). Gray points represent p-values of individual SNPs. The red line indicates the level of significance.

Of the 192 individuals genotyped on the Affymetrix 57K SNP chip, nine were removed because of low genotyping rate, leaving 64 R x R and 119 A x A individuals. After filtering for unaligned (1,844) and fixed (A x A = 23,383 (40.67%); R x R = 32,880 (57.18%)) markers within each dataset, a total of 32,274 markers were retained for analysis in the A x A cross and 22,777 markers were retained in the R x R cross. Following the FDR correction on the p-value, 4,994 markers (~15%) were significantly associated with life history within the A x A cross, and 436 markers (~2%) were significantly associated with life history within the R x R cross (FDR corrected p-value < 0.01). In the A x A cross, significant markers were located on all 29 *O*. *mykiss* chromosomes, while in the R x R cross there were significant markers on every chromosome except for chromosome 18. Although the sliding window analysis suggested that there were not any regions of the genome with an increased number of significant SNPs, both Omy06 and Omy25 appeared to be slightly enriched for associated markers in the A x A cross compared to the rest of the genome (Fig 1). No regions stood out as having an increase in the number of associated SNPs in the R x R cross (Fig 1).

### Genome alignment and marker annotation

In the A x A cross, twenty-nine genes that contained alleles significantly associated with migration were assigned to one of five smoltification-related functional groups: apoptosis (3 genes), phototransduction (3 genes), lipid and fatty acid metabolism (4 genes), growth and development (11 genes), and ion homeostasis (8 genes). These processes have all been shown to be connected to smoltification [42, 43], and previous work in the Sashin system has found differential expression of multiple genes within these five categories between cross types and life history strategies ([33, 40, 44]; see Table 1 for more details on the identity and function of these genes). Five genes on three chromosomes contained SNPs significantly associated with migration in both the A x A and R x R crosses, though none of those genes had functions known to be related to smoltification. No significant markers were located in genes with obvious smoltification-related functions in the R x R samples.

**Table 1:** Genes in A x A cross with functions related to smoltification and that encompass one or more SNPs associated with life history variation.

	Chromosome	Position (MB)	Annotation
**Apoptosis**	omy06	82.137	death-associated protein kinase 2
	omy07	78.259	programmed cell death 2-like
	omy09	54.514	caspase 9
**Phototransduction**	omy04	0.679	stimulated by retinoic acid 6
	omy16	1.946	opsin 5
	omy27	1.547	S-antigen; retina and pineal gland (arrestin)
**Lipid/Fatty Acid Metabolism**	omy02	53.713	cholesteryl ester transfer protein, plasma
	omy08	9.245	apolipoprotein B
	omy09	51.478	proteolipid protein 2
	omy16	52.253	lipopolysaccharide binding protein
**Growth and Development**	omy01	14.034	Janus kinase 2
	omy01	61.470	thyroid adenoma associated
	omy02	74.185	cortactin binding protein 2
	omy04	0.679	stimulated by retinoic acid 6
	omy07	11.474	cathepsin S
	omy08	76.874	purine nucleoside phosphorylase
	omy14	59.706	cathepsin O
	omy21	44.434	methyltransferase like 12
	omy22	28.173	fibroblast growth factor 14
	omy22	32.457	myosin, light chain 1, alkali; skeletal, fast
	omy28	11.228	epidermal growth factor receptor
**Ion Homeostasis**	omy01	52.900	SPARC related modular calcium binding 2
	omy01	55.613	transmembrane protein 72
	omy07	72.345	solute carrier family 39, member 8
	omy09	44.791	catenin, beta interacting protein 1
	omy22	2.791	sodium leak channel, non-selective
	omy22	47.354	transmembrane protein 237
	omy23	9.020	calcium homeostasis modulator 3
	omy28	59.52	solute carrier family 26, member 5

### Population genetic metrics

**Fig 2:**
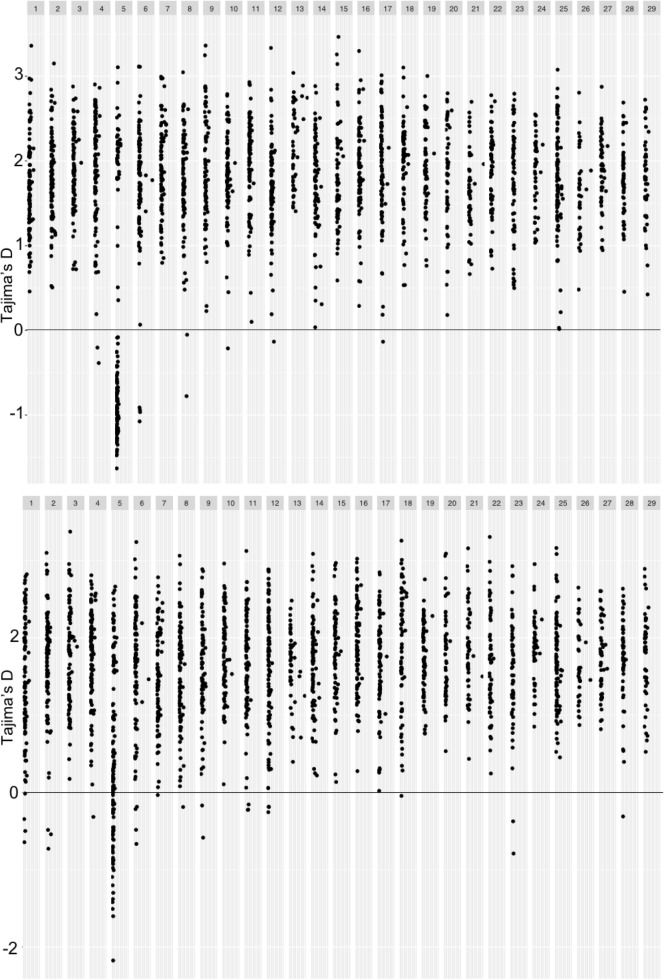
Tajima’s D estimates from both A x A (top panel) and R x R (bottom panel).

**Fig 3:**
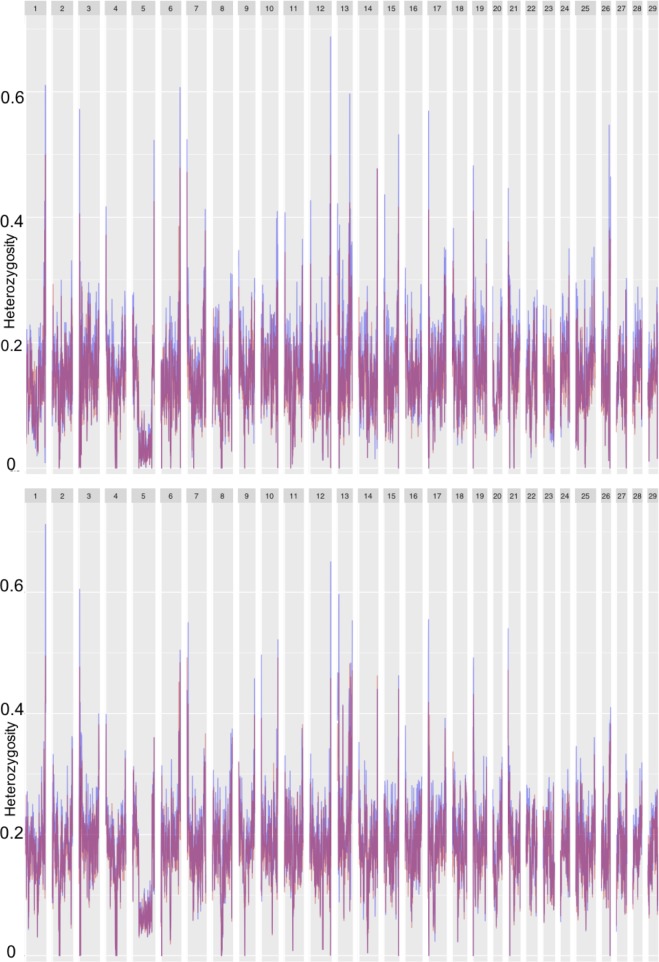
Observed (blue) and expected (red) heterozygosities for the A x A samples (top panel) and R x R samples (bottom panel). Sliding window averages are reported.

**Fig 4:**
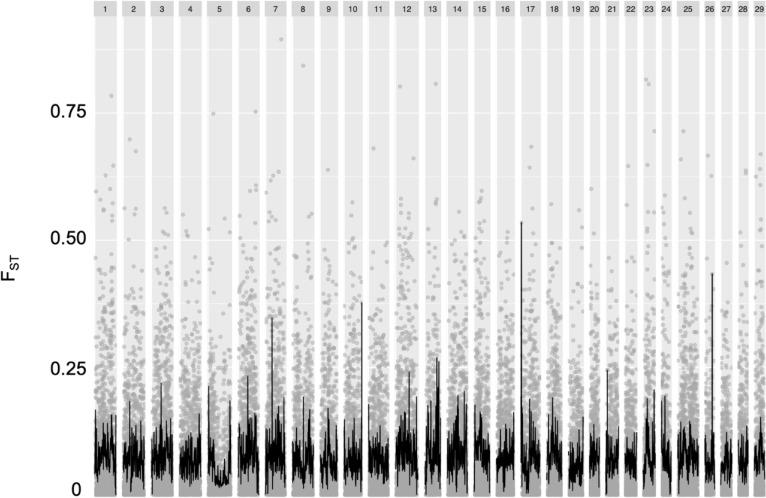
Sliding window analysis of *F*_*ST*_ outliers between A x A and R x R samples. Gray points represent *F*_*ST*_ estimates for individual SNPs, and the black line is the sliding window average.

We performed sliding window analyses on Tajima’s D (Fig 2), *H*_*O*_ and *H*_*S*_ (Fig 3), and *F*_*ST*_ (Fig 4). Chromosome 5, which displayed distinctive trends across these metrics, will be subsequently discussed in depth. Overall, however, we saw a positive trend in Tajima’s D (Fig 2), suggesting the signatures of either balancing selection or a recent population expansion in both cross types; average Tajima’s D values were 1.93 for the R x R cross and 1.98 for the A x A cross. Mean observed and expected heterozygosities were 0.181 (*H*_*O*_) and 0.171 (*H*_*S*_) for the A x A cross and 0.148 (*H*_*O*_) and 0.137 (*H*_*S*_) for the R x R cross, respectively. Along with an overall elevated *H*_*O*_ as compared to *H*_*S*_, there were individual points along several chromosomes (Fig 3) where *H*_*O*_ was greater, indicating more genetic variation than expected at those points. Notable peaks within the A x A samples included chromosomes 1, 3, 12, and 13. Similarly, peaks of observed heterozygosity were noted at chromosomes 1, 12, and 13 in the R x R samples. For the *F*_*ST*_ analysis we treated each cross as a population, which allowed us to evaluate differentiation between individuals with parents from the creek and those with parents from the lake. *F*_*ST*_ values at individual markers ranged from zero to 0.89, while the average *F*_*ST*_ within each sliding window ranged from zero to 0.53. The overall mean *F*_*ST*_ was 0.086. Regions of elevated *F*_*ST*_, indicating divergence between the two populations, were seen on chromosomes 7, 10, 17, and 26 (Fig 4).

We identified 38 outlier *F*_*ST*_ markers between the two crosses. These outlier SNPs were located on 20 chromosomes (FDR corrected P-value <0.05; Table 2), suggesting that the targets of selection are varied and found on multiple chromosomes between the two cross types. Although outliers were distributed on the majority of chromosomes, there did seem to be slight enrichment on chromosome 12 (5 outliers). However, these five outliers were distributed throughout the chromosome from 21.4 MB to 70.9 MB, suggesting there was not a specific area on chromosome 12 subjected to selection. Overall, there did not seem to be a strong consensus as to the function of the genes that included outliers, however, some were connected to immunity, suggesting the possibility of balancing or positive selection as a result of contact with different pathogens between the two populations.

**Table 2:** Significant *F*_*ST*_ outliers between the A x A and R x R samples together with positional information on the rainbow trout genome and annotation of the gene the allele was found within. N/A signifies that the outlier was not in a known protein-coding gene.

SNP ID	Chromosome	Position (MB)	F_ST_	FDR-corrected P-value	Gene Annotation
88936105	1	22.445	0.444	0.018	heat shock protein
88957602	1	35.521	0.438	0.026	V(D)J recombination-activating protein 1-like
88955418	1	35.54	0.438	0.027	V(D)J recombination-activating protein 1-like
88951075	2	26.994	0.470	0.006	NADP-dependent malic enzyme-like
88957040	2	51.038	0.465	0.007	N/A
88914811	3	45.891	0.417	0.049	ly6/PLAUR domain-containing protein 1-like
88911072	3	53.165	0.423	0.046	lunapark-A-like
88919751	3	60.337	0.434	0.026	disrupted in renal carcinoma protein 2-like
88914847	4	13.125	0.432	0.036	NUAK family SNF1-like kinase 1
88941338	4	25.675	0.425	0.039	cep135
88942593	5	21.468	0.485	0.002	N/A
88942168	7	25.052	0.432	0.032	receptor-type tyrosine-protein phosphatase T-like
88955181	7	62.371	0.496	0.004	lymphoid-restricted membrane protein-like
88930010	8	43.076	0.506	0.001	N/A
88907598	8	76.6	0.438	0.023	succinate dehydrogenase cytochrome b560 subunit, mitochondrial-like
88959735	10	29.114	0.434	0.034	WSC domain-containing protein 1-like
88935631	10	33.381	0.443	0.014	MICOS complex subunit MIC27-like
88938075	11	19.465	0.466	0.008	noelin-3-like
88941059	12	21.423	0.430	0.031	myosin-XVIIIa-like
88922172	12	21.528	0.445	0.016	CUE domain-containing protein 1-like
88943537	12	39.125	0.425	0.043	opioid-binding protein/cell adhesion molecule-like
88958502	12	53.073	0.434	0.034	rabankyrin-5-like
88924335	12	70.959	0.464	0.008	erythropoietin receptor-like
88945138	14	46.599	0.437	0.021	PPARG coactivator 1 beta
88915460	15	22.921	0.426	0.037	tmx3 thioredoxin related transmembrane protein 3
88914640	17	41.398	0.469	0.005	SOSS complex subunit B1-like
88932095	19	42.646	0.435	0.030	fibronectin type III domain-containing protein 5-like
88914058	19	54.384	0.425	0.042	rpusd2 RNA pseudouridylate synthase domain containing 2
88957386	20	17.719	0.421	0.045	proto-oncogene Wnt-3
88905382	22	7.778	0.437	0.028	mitd1 microtubule interacting and trafficking domain containing 1
88940367	22	13.828	0.461	0.011	nepro nucleolus and neural progenitor protein
88925039	23	12.494	0.496	0.001	N/A
88959827	23	17.020	0.423	0.040	serine-rich coiled-coil domain-containing protein 2-like
88923218	23	22.817	0.417	0.019	inorganic pyrophosphatase-like
88905936	26	12.712	0.464	0.009	RING finger protein 166
88942569	28	31.119	0.459	0.010	septin-2-like
88906826	28	31.120	0.460	0.012	septin-2-like
88924385	29	22.781	0.422	0.048	mcc MCC, WNT signaling pathway regulator

Linkage disequilibrium was calculated separately for each cross type and each chromosome, and the R x R cross type had consistently higher LD in every chromosome–except for chromosome 5 –when compared to the A x A cross type suggesting a lower amount of genetic diversity in the Lake population. Average intrachromosomal LD was high in both cross types (0.339 in the A x A cross and 0.375 in the R x R cross; see S2 Table), which is not surprising given both the limited number of informative meiosis possible when performing crosses and the small population size of both populations [32].

### Evidence for the inversion on Omy05

**Fig 5:**
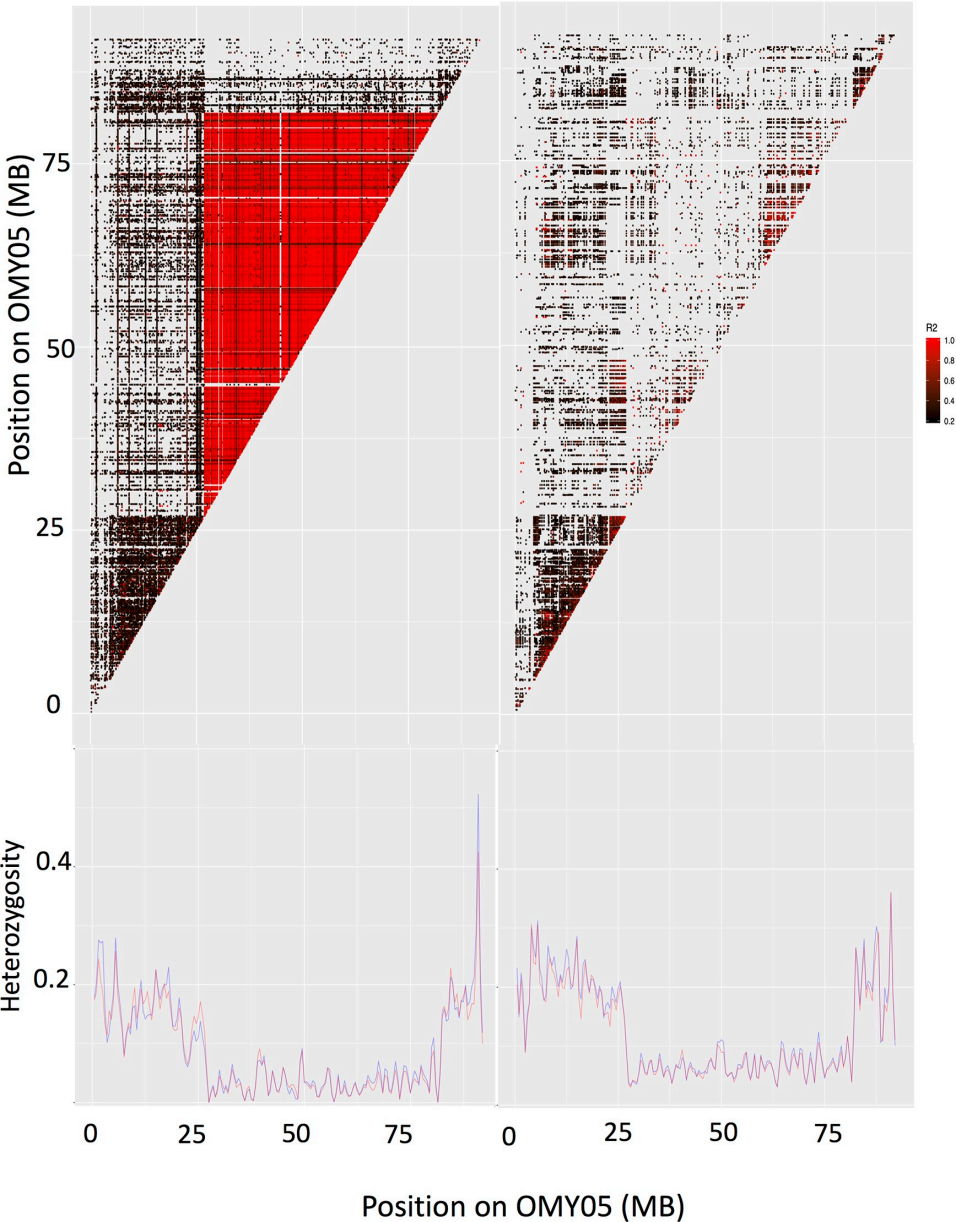
Linkage disequilibrium heatmap constructed from r^2^ values for the A x A samples (left) and the R x R samples (right) within chromosome 5 (top panel) and observed and expected heterozygosities for the A x A samples (left) and the R x R samples (right) within chromosome 5 (bottom panel).

One region spanning most of chromosome 5 stood out across several population genetic metrics, namely observed and expected heterozygosity and Tajima’s D (Figs 2 and 3). This region was characterized by low observed and expected heterozygosity, and there was an overall negative trend in Tajima’s D as compared to the rest of the genome. This region was also associated with high linkage disequilibrium (Fig 5), notably in the A x A cross type. Contrasted to trends across the rest of the genome, the A x A cross had higher LD (average r^2^ value of 0.857) on chromosome 5 than did the R x R cross (average r^2^ value of 0.350; S2 Table; Fig 5), likely due to a reduction in genetic variation in R x R samples compared to the A x A samples.

In the A x A families, the inversion spans from ~ 27 MB to ~ 81.8 MB (Fig 5). Inspection of individuals’ genotypes within this region found that 10 A x A and zero R x R samples were heterozygous for the inversion–i.e., having one ancestral chromosome and one derived chromosome–as determined by exclusively heterozygous SNPs in the inverted region in those 10 samples. All other samples were classified as derived, and no individual was homozygous for the ancestral form of the inversion (S1 Table). Nine heterozygous samples were phenotyped as smolts and one was classified as a resident, though it is important to note that the resident sample could have undergone smoltification after the individuals were phenotyped. Although this may suggest a link between having a copy of the ancestral form of the inversion and anadromy, 64 A x A individuals with two copies of the derived form of the inversion (87.7%) were classified as migratory smolts, suggesting that any association between the anadromous copy of the inversion and a migratory life history is limited. Furthermore, we found no statistical association between the inversion and life history development (*X*^*2*^ = 3.78, p > 0.05). The presence of the ancestral form of the inversion at low frequencies in Sashin Creek is also supported by a reanalysis of the RAD-seq data published in Hale et al. 2013 [29]. This reanalysis found no evidence for the ancestral form of the inversion in the lake population, and an approximately 5% frequency of the ancestral form in the migratory steelhead population in Sashin Creek. As with the data generated herein, the re-analyzed samples that had the ancestral form of the inversion were all heterozygous for the inversion (MC Hale, unpublished data).

## Discussion

Identifying the alleles behind the development of parallel phenotypic change is a key area of research in evolutionary biology. One of the best studied examples of parallel genetic adaptation is the repeated occurrence of freshwater residency in threespine sticklebacks, where hard selective sweeps are associated with the rapid colonization of freshwater environments from marine ancestors (e.g., [4, 5, 45]). In contrast, we have a limited understanding of the genetic architecture of parallel phenotypic adaptation when the phenotype in question is quantitative in nature, physiologically and behaviorally complex, and highly influenced by the environment.

Life history development in salmonids is a classic example of a quantitative trait that is determined by complex interactions between genetic and environmental effects. However, while life history development in rainbow trout exhibits parallel phenotypic change (i.e., native populations throughout the range of the species produce both migrants and residents), the alleles associated with this trait are not shared (e.g., compare results in [28] to [29]). For example, although some populations of rainbow trout show a strong association between a large-scale inversion on chromosome Omy05 and life history development, the data presented herein indicates that this association is not found in Sashin Creek. Moreover, a similar lack of association between the inversion and life history has been found in other high latitude populations of rainbow trout [12, 25], in which individuals have the derived inversion haplotype at high frequencies regardless of the life history of the individual. While this could suggest a reduction in the influence of genetics on life history determination in these populations, we believe this is not the case as previous studies in Sashin Creek suggest that life history development has a strong genetic component [27, 29, 30, 32]. Consequently, it appears that life history development in the Sashin Creek system is regulated by polymorphisms in regions of the genome outside of the Omy05 inversion.

Although the association of the inversion with life history development seems to disappear with increased latitude, we suggest that this region of the genome encodes other traits that are essential for survival and adaptation in high-latitude rainbow trout. One trait that has been consistently associated with this region of Omy05 is embryonic development rate (e.g., [17, 21, 22, 46]). For example, Miller et al. [22] reported that two different populations of rainbow trout–Swanson (derived from a resident population from Alaska) and Clearwater (derived from an anadromous population from Idaho)–had alleles associated with fast development rate, presumably allowing individuals adapted to cold water to hatch early and take advantage of the brief northern summer. These clonal lines were both from cold water areas, and were both founded by individuals homozygous for the derived form of the inversion. Although development rate was not tested in the samples used herein, a previous study in the Sashin population found a significant difference in embryonic development rate between samples produced from Sashin Lake (i.e., R x R) and Sashin Creek (i.e., A x A; [46]), with the R x R samples developing faster. These results support what is reported herein, suggesting that the inversion is associated with phenotypes other than life history development. If the inversion is in fact associated with development rate, then selection for the derived form of the inversion would increase in frequency in areas where fast development rate is advantageous (i.e., in northern populations with a reduced window for growth). Further studies that categorize the frequency of the inversion at the population level and studies that investigate associations between the inversion and development rate as well as the association between the inversion and life history development are required.

Ultimately, the distribution of the different inversion haplotypes may be due to the re-colonization of northern areas after the recession of the Cordilleran ice sheet ~10,000 years ago. At its maximum extent the Cordilleran ice sheet covered much of the northern range of rainbow trout, including northern Washington, British Colombia, and southern Alaska. The occurrence of both forms of the inversion in northern latitudes suggests either i) that re-colonization included trout with both inversion haplotypes [25], and/or ii) that northern latitudes were re-colonized mainly by non-anadromous individuals (with the derived version of the inversion) that survived the last glaciation in an inland refugia [47]. However, if, as seems likely, the migratory rainbow trout that recolonized northern systems did possess both forms of the inversion, then this would provide additional evidence that the inversion is not associated with life history. Instead, we propose that residency in northern latitudes has evolved from standing genetic variation in a population-specific manner. The derived form of the inversion was then strongly selected for where cold freshwater environments, such as Sashin Creek, were utilized for spawning. A similar result was reported in Arostegui et al. [25] who found higher frequency of the inverted condition in stream compared to lake populations of rainbow trout. Presumably, streams will be colder than lakes as small bodies of water lose heat faster than large bodies. As stated above, further studies from other northern latitude populations of rainbow trout will help confirm the role of the inversion in life history development versus other phenotypes such as cold-water adaptation and development rate.

### Genomic association with juvenile migration

As anadromous migration requires many changes to the physiology, behavior, and morphology of an individual, it is not surprising that the molecular basis of life history development is due to many alleles of small effect scattered throughout the genome, a pattern found in studies across the range of rainbow trout (e.g., [26, 28, 29]). However, although these studies confirm a genetic basis to migration, the alleles associated with life history development are different, suggesting population-specific genetic effects. These population-level differences may be explained by repeated independent selection on different alleles from standing genetic variation. Support for this conclusion comes from studies that have compared relatedness between individuals from different drainages. In all studies, individuals of different life histories (i.e., migrant and resident) within a drainage system are more closely related to each other than to individuals of the same life history from different populations [48, 49]. Thus, many individuals have the ability to respond to environmental cues and adopt the alternative life history to their familial origin. This is confirmed by the data presented herein, as both cross types produced residents, sexually mature males, and migratory smolts, as has consistently been found in manipulated crosses in the Sashin system [27, 30, 32, 33].

Though the present study failed to find an association with loci on chromosome 5, we did find associations within genes that previous research has suggested might be important with life history development in the Sashin rainbow trout (Table 1). Perhaps most compelling, are the associations of alleles within genes involved with phototransduction, namely, Opsin 5 and Arrestin. Both of these genes have been found to be differentially expressed in the brains of Sashin Creek rainbow trout between A x A and R x R crosses during the first two years of development [33, 40]. Together these results suggest that polymorphisms within these genes are associated with their differential expression and the development of different migratory tactics. The differential expression of cathepsin genes have also found to be important in the smoltification process [50]. Alleles in Cathepsin S and Cathepsin O were found to be associated with life history development in the A x A samples (Table 1). Cathepsins have multiple roles including development, cell turnover, and growth. These, as well as the other genes specified in Table 1 provide good candidates for further experiments. However, it is important to emphasize that the large number (4,994) of markers associated with life history development, and our F_ST_ outlier analysis (Table 2), suggests that the proximate molecular mechanisms and the ultimate evolutionary processes promoting life history development, are complex and varied.

One of the most notable results from our GWAS was the differences in the number of genes associated with life history development between the A x A and R x R populations. Although this might suggest the genetic basis for life history development is different between samples in Sashin Creek and Sashin Lake an alternative prediction is that these differences are caused by a lack of genetic variation in the lake population. As already stated, the lake population was founded by transplanting fish from the creek above two barrier waterfalls in the 1920s. Since that event there have been no additional introductions [31]. Therefore, samples within the lake have been through a substantial genetic bottleneck as supported by lower observed and expected heterozygosity (Fig 3) and higher estimates of LD (S2 Table) in the R x R samples compared to the A x A samples. Although our sampling confirms that resident samples can produce migrants and v*ice versa*, it is important to note that previous studies have found that migrants produced by residents are less likely to return to spawn than migrants produced from migratory samples [31]. These differences between adjacent populations add to a growing body of literature that suggests life history development is controlled by population specific genetic effects.

## Conclusions

In this study, we identified a large-scale chromosomal inversion on Omy05 in the Sashin Creek population of rainbow trout. While both haplotypes are present in the population, the frequency of the ancestral type is low and is not associated with migration. This finding, together with similar studies in other high latitude populations of rainbow trout, suggests that the importance of the inversion with respect to life history development is population-specific rather than consistent across the range of rainbow trout, and it is probable that the inversion is associated with adaptive traits other than life history development.

It is important to note that in many of the previous studies that have documented strong evidence for an association between the inversion and life history development, there were both man-made and natural barriers that separated migratory individuals from residents. Although this situation is found in multiple populations across the range of rainbow trout, many other populations are typified by both phenotypes existing in sympatry. The association between the inversion and life history development in these populations is largely unknown and we propose that further studies in such populations may be informative. While the genetic basis of migration in salmonids is complex and, likely, population specific, studies such as these increase our understanding of the inheritance of quantitative traits, and the advancement of genetic methods that allow for thousands of markers to be genotyped allows us to better study and understand partial migration as a whole.

## Supporting information

S1 Table SNP names, genotypes and positional information for all samples genotyped using the 57K rainbow trout SNP chip (described in Palti et al. 2015). (ZIP)

S2 Table Average measurements of linkage disequilibrium (r2) within each chromosome. Both the R x R and A x A samples are shown.
(DOCX)
