# Supplementary material for: A large-scale chromosomal inversion is not associated with life history development in rainbow trout from Southeast Alaska
Source: PLoS One. 2019 Sep 20;14(9):e0223018. doi: 10.1371/journal.pone.0223018 (PMC6754156; doi:10.1371/journal.pone.0223018)
Supplement: S2 Table — Both the R x R and A x A samples are shown. (DOCX) [file pone.0223018.s002.docx]

S2 Table: Average measurements of linkage disequilibrium (r^2^) within each chromosome

| chromosome | A x A | R x R |  |  |
| --- | --- | --- | --- | --- |
| 1 | 0.322 | 0.384 | | |
| 2 | 0.313 | 0.362 | | |
| 3 | 0.316 | 0.367 | | |
| 4 | 0.315 | 0.356 | | |
| 5 | 0.857 | 0.370 | | |
| 6 | 0.325 | 0.396 | | |
| 7 | 0.324 | 0.377 | | |
| 8 | 0.321 | 0.365 | | |
| 9 | 0.321 | 0.371 | | |
| 10 | 0.324 | 0.355 | | |
| 11 | 0.315 | 0.368 | | |
| 12 | 0.324 | 0.380 | | |
| 13 | 0.315 | 0.385 | | |
| 14 | 0.326 | 0.362 | | |
| 15 | 0.320 | 0.386 | | |
| 16 | 0.317 | 0.372 | | |
| 17 | 0.319 | 0.382 | | |
| 18 | 0.315 | 0.390 | | |
| 19 | 0.318 | 0.358 | | |
| 20 | 0.315 | 0.381 | | |
| 21 | 0.320 | 0.372 | | |
| 22 | 0.317 | 0.363 | | |
| 23 | 0.323 | 0.390 | | |
| 24 | 0.321 | 0.376 | | |
| 25 | 0.329 | 0.366 | | |
| 26 | 0.326 | 0.395 | | |
| 27 | 0.324 | 0.394 | | |
| 28 | 0.322 | 0.356 | | |
| 29 | 0.317 | 0.396 | |  |
